# Deep learning-based automatic pipeline for 3D needle localization on intra-procedural 3D MRI

**DOI:** 10.1007/s11548-024-03077-3

**Published:** 2024-03-23

**Authors:** Wenqi Zhou, Xinzhou Li, Fatemeh Zabihollahy, David S. Lu, Holden H. Wu

**Affiliations:** 1 Department of Radiological Sciences, University of California Los Angeles, 300 UCLA Medical Plaza, Suite B119, Los Angeles, CA 90095 USA; 2 Department of Bioengineering, University of California Los Angeles, Los Angeles, CA USA; 3 Joint Department of Medical Imaging, Sinai Health System and University of Toronto, Toronto, Canada

**Keywords:** Interventional MRI, Device tracking, Transformer networks, Deep learning, Image segmentation, Needle feature

## Abstract

**Purpose:**

Accurate and rapid needle localization on 3D magnetic resonance imaging (MRI) is critical for MRI-guided percutaneous interventions. The current workflow requires manual needle localization on 3D MRI, which is time-consuming and cumbersome. Automatic methods using 2D deep learning networks for needle segmentation require manual image plane localization, while 3D networks are challenged by the need for sufficient training datasets. This work aimed to develop an automatic deep learning-based pipeline for accurate and rapid 3D needle localization on in vivo intra-procedural 3D MRI using a limited training dataset.

**Methods:**

The proposed automatic pipeline adopted Shifted Window (Swin) Transformers and employed a coarse-to-fine segmentation strategy: (1) initial 3D needle feature segmentation with 3D Swin UNEt TRansfomer (UNETR); (2) generation of a 2D reformatted image containing the needle feature; (3) fine 2D needle feature segmentation with 2D Swin Transformer and calculation of 3D needle tip position and axis orientation. Pre-training and data augmentation were performed to improve network training. The pipeline was evaluated via cross-validation with 49 in vivo intra-procedural 3D MR images from preclinical pig experiments. The needle tip and axis localization errors were compared with human intra-reader variation using the Wilcoxon signed rank test, with *p* < 0.05 considered significant.

**Results:**

The average end-to-end computational time for the pipeline was 6 s per 3D volume. The median Dice scores of the 3D Swin UNETR and 2D Swin Transformer in the pipeline were 0.80 and 0.93, respectively. The median 3D needle tip and axis localization errors were 1.48 mm (1.09 pixels) and 0.98°, respectively. Needle tip localization errors were significantly smaller than human intra-reader variation (median 1.70 mm; *p* < 0.01).

**Conclusion:**

The proposed automatic pipeline achieved rapid pixel-level 3D needle localization on intra-procedural 3D MRI without requiring a large 3D training dataset and has the potential to assist MRI-guided percutaneous interventions.

**Supplementary Information:**

The online version contains supplementary material available at 10.1007/s11548-024-03077-3.

## Introduction

Image-guided percutaneous interventions play key roles in cancer diagnosis and treatment with their minimally invasive characteristics [1, 2]. Currently, most percutaneous interventions are guided by ultrasound (US) or computed tomography (CT) [3, 4]. However, US and CT suffer from insufficient soft-tissue contrast and poor visibility of several important classes of tumors [5, 6]. In comparison, magnetic resonance imaging (MRI) provides excellent soft-tissue contrast and can be the only modality for visualizing tumors that are not visible on CT or US, which makes it an emerging imaging modality for guiding percutaneous interventions in various applications including needle-based targeted biopsy and focal ablation in the liver or other abdominal organs [7–11].

Despite the advantages of MRI-guided interventions, accurate and rapid 3D localization of the interventional needle in intra-procedural MR images remains a major challenge [12, 13]. The interventional needle can be visualized on MR images based on the passive signal void feature caused by needle-induced susceptibility effects [14]. In current workflows, 3D needle localization is performed manually; interventional radiologists locate the needle by marking the needle entry point and tip on the intra-procedural 3D MR images [15]. However, manual needle localization requires expert knowledge and is time-consuming, which leads to cumbersome workflows, prolonged procedure time, and potential variability [16, 17]. The lack of timely feedback regarding needle and target locations also hinders the possibility of real-time MRI-guided interventions under human operation or with robotic assistance [18, 19].

2D deep learning networks for automatic 2D needle segmentation and localization have shown promising results [20–22]. However, 2D needle localization methods commonly require the initial manual localization of a 2D image plane that contains the needle feature [23]. Moreover, the 2D needle localization results do not immediately provide the essential 3D relative positions of the needle and the target needed for guiding needle insertion. On the other hand, training 3D deep learning networks for needle segmentation on MR images typically requires large 3D training datasets [24], which may not be available for specific MRI-guided procedures or at specific facilities. Studies of applying 3D deep learning networks for needle segmentation on CT and US images have similarly demonstrated the data-demanding nature of these networks [25, 26]. The potentially limited sizes of intra-procedural 3D MR image datasets and the variabilities in the needle feature's location and grayscale appearance in in vivo 3D MRI may lead to insufficient training of the 3D deep learning network and result in inaccurate 3D needle feature segmentation and localization.

For the task of 3D needle segmentation, which requires delineating a relatively small object in a large field-of-view (FOV), convolutional neural network (CNN)-based 3D neural networks may be suboptimal since the convolution operations lack the ability to efficiently capture global information [27, 28]. To better model the long-range information in large FOVs, researchers have developed transformer-based networks that adopt the self-attention mechanism to capture global interactions between contexts [29]. The Shifted Window (Swin) Transformer introduced by Liu et al. demonstrated excellent results with its hierarchical architecture, which enables the model to capture both local and global information [30]. In the context of 3D medical image segmentation, Hatamizadeh et al. further introduced the 3D Swin UNEt TRansformer (UNETR) [31] which utilized a U-shaped network structure with a Swin Transformer-based encoder and CNN-based decoder. Researchers demonstrated the efficacy of Swin UNETR in 3D medical image segmentation in the BraTS 2021 segmentation challenge, where it outperformed UNet and nnU-Net [31].

In this work, our objective was to develop an automatic pipeline for rapid and accurate 3D needle localization on 3D MRI by taking advantage of transformer networks. To overcome the restriction of limited 3D datasets for training, we combined the 3D Swin UNETR and 2D Swin Transformer for coarse-to-fine segmentation and adopted pre-training and data augmentation strategies. The proposed pipeline was evaluated using in vivo 3D MR images acquired during MRI-guided liver interventions in preclinical pig models and compared with manual localization of the 3D needle feature.

## Methods

### MRI-guided interventional experiments

In an animal research committee-approved study, we performed MRI-guided targeted needle placement in the livers of seven healthy female pigs on a 3 T scanner (MAGNETOM Prisma, Siemens, Erlangen, Germany). These experiments were designed and performed by an experienced interventional radiologist (over 20 years of experience) based on step-and-shoot workflows that mimic clinical image-guided procedures at our institution [32–36].

**Fig. 1:**
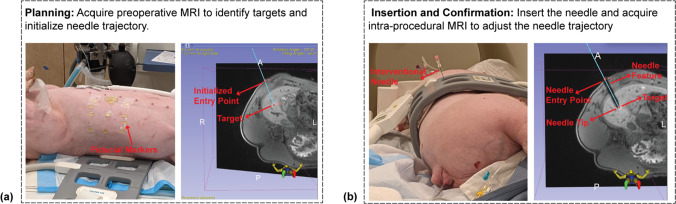
Manual needle localization workflow for preclinical MRI-guided percutaneous interventions. **a** Planning: Acquire preoperative MR images to localize targets and initialize needle entry point and trajectory. **b** Insertion and Confirmation: Insert the needle and adjust the needle trajectory based on intermediate confirmation scans until the needle tip reaches the target. Note that needle adjustment/insertion was performed with the subject table moved out of the MRI scanner bore

The workflow of the experiments is shown in Fig. 1. In the planning stage, preoperative 3D T_1_-weighted (T1w) gradient echo (GRE) Dixon MR images were acquired to localize the target and initialize the needle entry point and trajectory. In the insertion and confirmation stage, manual needle localization was performed by marking the needle entry point and needle tip on the 3D T1w GRE images in a graphical environment (3D Slicer) [37]. The patient table was moved out from the scanner for the interventional radiologist to insert and adjust the needle based on the 3D relative position of the needle tip and the target determined from MRI. This process was repeated until the needle tip reached the target.

### Intra-procedural MRI datasets

Intra-procedural 3D T1w GRE Dixon MR images containing the needle feature and 2D real-time golden-angle (GA) ordered radial GRE images with the image plane aligned with the needle axis were collected during experiments with parameters shown in Table 1. In each experiment, 7 3D T1w GRE images were acquired as confirmation images between needle adjustment steps, with the needle inserted at different depths and angles. Based on the needle location in the 3D confirmation images, 2D real-time GA ordered radial GRE images were acquired on manually located 2D oblique axial and sagittal planes aligned with the needle axis. In each experiment, 70 2D image frames with different insertion depths and angles were selected from the multiple real-time scans to form the 2D radial GRE dataset. Under the guidance and supervision of the interventional radiologist, a trained researcher manually annotated the needle feature on the 2D radial GRE images and 3D T1w GRE images to serve as reference segmentation masks. The 3D needle tip and axis references were annotated on the 3D T1w GRE images by marking the 3D coordinates of the needle tip and entry point. The 3D needle tip and axis annotation process were performed twice with a washout period of two weeks in between to assess the human intra-reader variation.

**Table 1:** MRI datasets and imaging parameters

	3D T1w Cartesian GRE dataset	2D radial GRE dataset
TR/TE (ms)	3.91/1.23 [OP], 2.46 [IP]	3.8/1.72; 5.08/3; 6.3/2.85
FOV	237 × 346 × 180 mm^3^	300 × 300 mm^2^
Number of slices	120	1
In-plane resolution	1.35 × 1.35 mm^2^	1.56 × 1.56 mm^2^
In-plane matrix size	176 × 256	192 × 192
Slice thickness	1.5 mm	5 mm
Flip angle	9°	9°
Parallel imaging factor	4	N/A
Acquisition time	13 s (breath held)	100 ms (breathing)
Size of dataset	49 3D volumes for cross-validation (7 3D volumes from each experiment)	490 2D images for cross-validation (70 2D images from each experiment)

*TR* repetition time, *TE* echo time, *OP* out of phase, *IP,* in phase, *FOV* field-of-view, *N*/*A* not applicable

### Automatic 3D needle localization pipeline

**Fig. 2:**
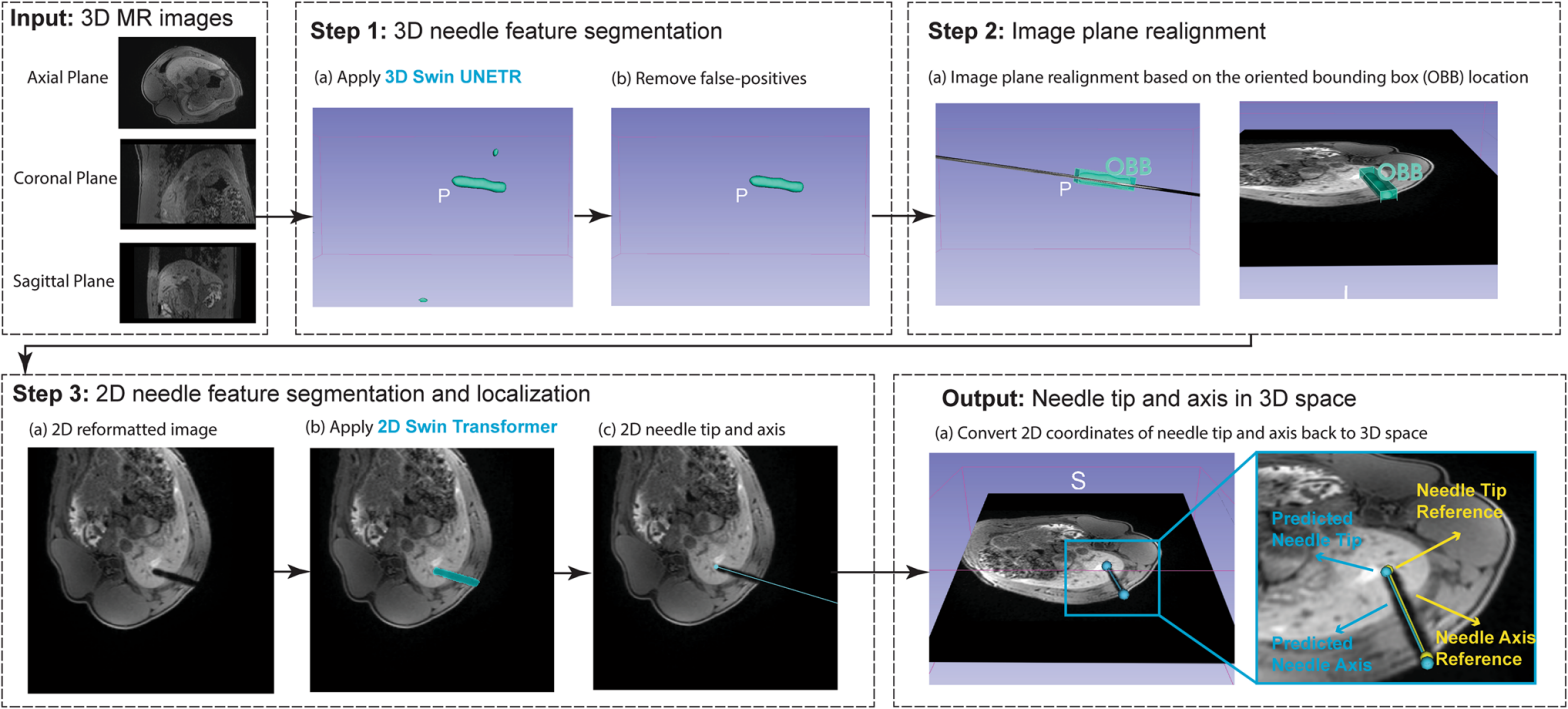
Diagram of the proposed pipeline. Input: 3D T1w GRE Dixon water images. Step 1: Apply the 3D Swin UNETR for initial 3D needle feature segmentation. Step 2: 2D oblique axial image plane realignment. Step 3: Apply the 2D Swin Transformer on the reformatted 2D image and localize the needle tip and axis in 2D. Output: Convert the 2D coordinates of the needle tip and axis back to 3D space for 3D visualization

We proposed a pipeline (Fig. 2) that takes 3D GRE images as input and localizes the needle feature tip and axis in 3D space via a fully automatic process. There were three main steps in the pipeline. Step 1: the 3D Swin UNETR was applied to the 3D GRE input images and generated the initial 3D needle feature segmentation. The 3D segmentation output was post-processed by a false-positive removal module which calculated the volume of each segmentation object and removed the small ones, as the needle segmentation object had the largest volume compared to the false positives caused by other regions of susceptibility or signal void in the body. Note that false positives connected to the needle segmentation object cannot be removed by this false-positive removal module. Step 2: we performed oblique axial image plane realignment along the main axis of the 3D segmentation output to generate a 2D reformatted image that contains the needle feature. Step 3: the 2D Swin Transformer network was applied to the 2D reformatted image to generate a 2D needle feature segmentation. We localized the 2D needle axis with orthogonal distance regression (ODR) [38]. The intersection of the 2D needle axis and the 2D segmentation mask was identified as the 2D needle feature tip and entry point [20]. We then converted the 2D coordinates of the needle tip and entry point into 3D based on the 2D reformatted image plane position.

To evaluate the necessity of the 2D Swin Transformer network, we compared the performance of the proposed pipeline with the pipeline without the 2D network (step 1 only), which identified the main axis of the 3D segmentation mask as the needle axis, and the intersection of the main axis and the surface of the 3D needle feature segmentation mask as the needle feature tip.

### Deep learning networks for needle feature segmentation

**Fig. 3:**
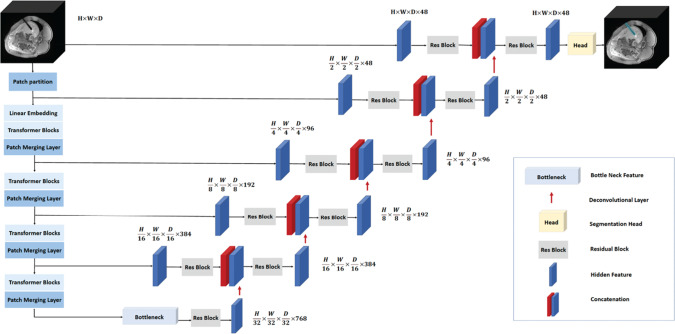
Overview of the 3D Swin UNETR architecture. The Swin UNETR processed 3D MR images as inputs and generated distinct patches from the input data to establish windows of various sizes for self-attention calculation. The Swin transformer's encoded feature representations were then transmitted to a CNN decoder through skip connections at various resolutions. W:256, H:256, D:128

**Fig. 4:**
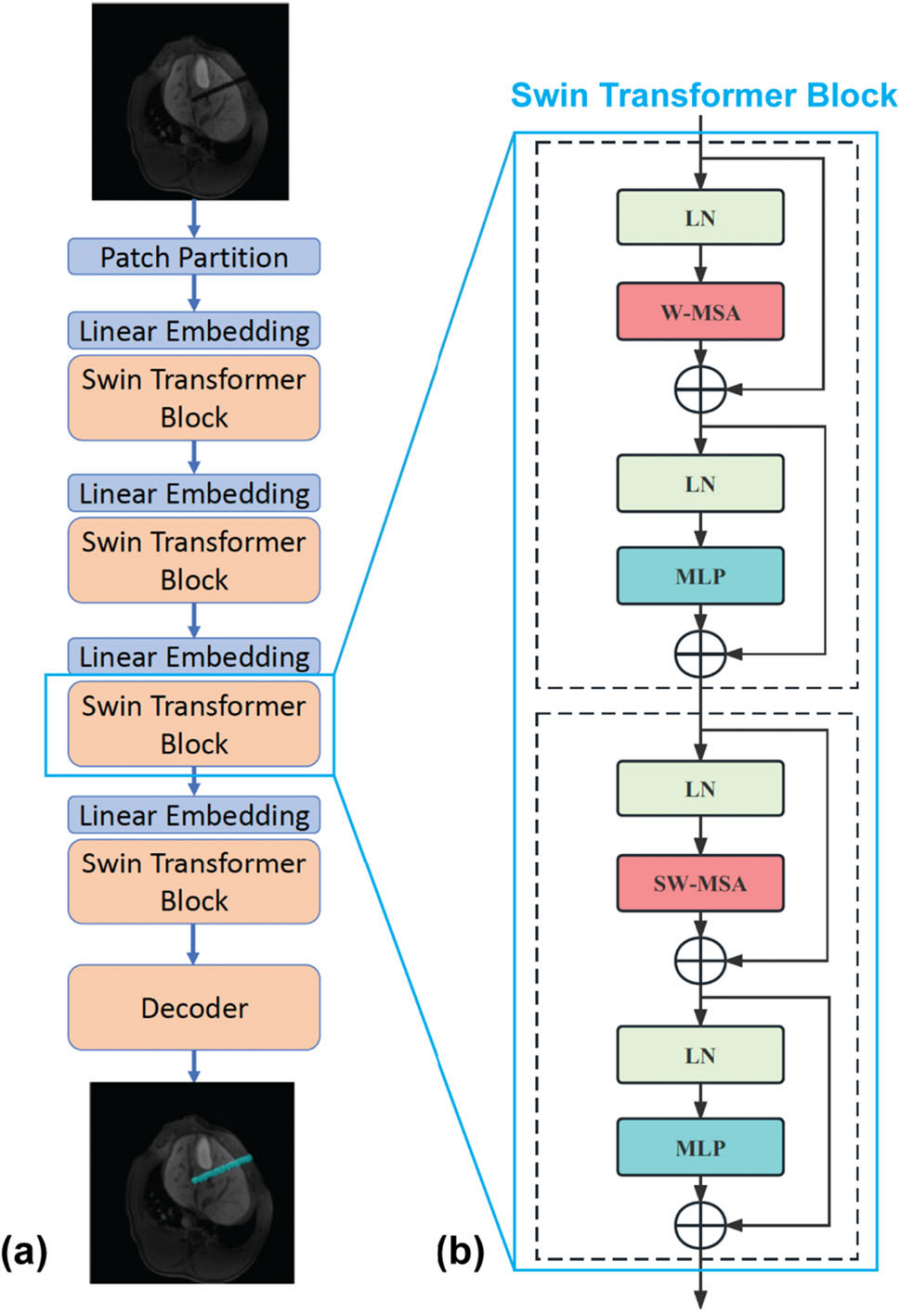
Overview of the 2D Swin Transformer architecture. **a** The architecture, input 2D MR image, and output 2D segmentation mask of the 2D Swin Transformer. **b** Two successive Swin Transformer Blocks. W-MSA and SW-MSA are multi-head self-attention modules with regular and shifted window configurations, respectively

We adopted the 3D Swin UNETR [31] (Fig. 3) with pre-trained weights generated by self-supervised learning tasks on publicly available unlabeled CT images of various human body organs without interventional needles [39] and fine-tuned the model using the intra-procedural 3D MR images. We pre-trained the 2D Swin Transformer [40] (Fig. 4) using 2D radial GRE images and then fine-tuned the network using the 2D reformatted images generated by step 2 in the pipeline. Fifteen-fold data augmentation was performed for the training process. To demonstrate the advantages of the 2D and 3D Transformer networks compared with the UNet, we trained 2D UNet and 3D UNet with the same dataset and cross-validation strategy for comparison. The hyperparameters and data augmentation details are shown in Table 2.

**Table 2:** Parameters for 3D (3D Swin UNETR and 3D UNet) and 2D neural networks (2D Swin Transformer and 2D UNet)

	3D UNet	3D Swin UNETR	2D UNet	2D Swin Transformer
Loss function	Dice loss		Dice loss	
Learning rate	0.001		Pre-training: 0.001 Fine-tuning: 0.00001	
Optimizer	Adam		Adam	
Batch size	4		8	
Number of epochs	200		Pre-training: 200 Fine-tuning: 100	
Trainable weights	4,808,917	62,186,708	1,625,161	48,962,404
Training time	~ 4 h	~ 5 h	~ 1 h	~ 2 h
Inference time	2.67 s	2.14 s	0.016 s	0.011 s
Data augmentation	15 fold: random rotation, flipping, translation, zooming, additive Gaussian noise			

All networks were implemented using Keras and PyTorch

### Evaluation metrics

To evaluate the needle feature segmentation performance of the 3D Swin UNETR and 2D Swin Transformer, 3D and 2D Dice scores (0–1) of the output segmentations before post-processing were calculated. For 3D needle feature tip and axis localization evaluation, the Euclidean distance between the predicted needle tip and reference needle tip ${(\varepsilon }_{tip })$ in mm and the angle between the predicted needle axis and needle axis reference $(\alpha )$ in degrees were calculated in 3D space. We performed seven-fold cross-validation using a total of 49 3D volumes (7 from each experiment), where each fold consisted of one experiment's images (7 3D volumes) as the validation set while the training set consisted of images collected from the six remaining experiments (42 3D volumes).

### Statistical analysis

We compared differences in the performance (Dice score) of the Swin Transformer-based networks and UNet-based networks, as well as 3D needle localization accuracy (tip and axis error) with and without the 2D Swin Transformer network. For experiments with more than two sets of data samples, the Kruskal–Wallis test was applied first; if the differences were significant across the sets, comparisons were then conducted between pairs of samples using the Wilcoxon signed rank test. Multiple comparisons were accounted for by using Bonferroni correction. A *p* < 0.05 was considered significant.

## Results

### 3D and 2D needle feature segmentation

**Fig. 5:**
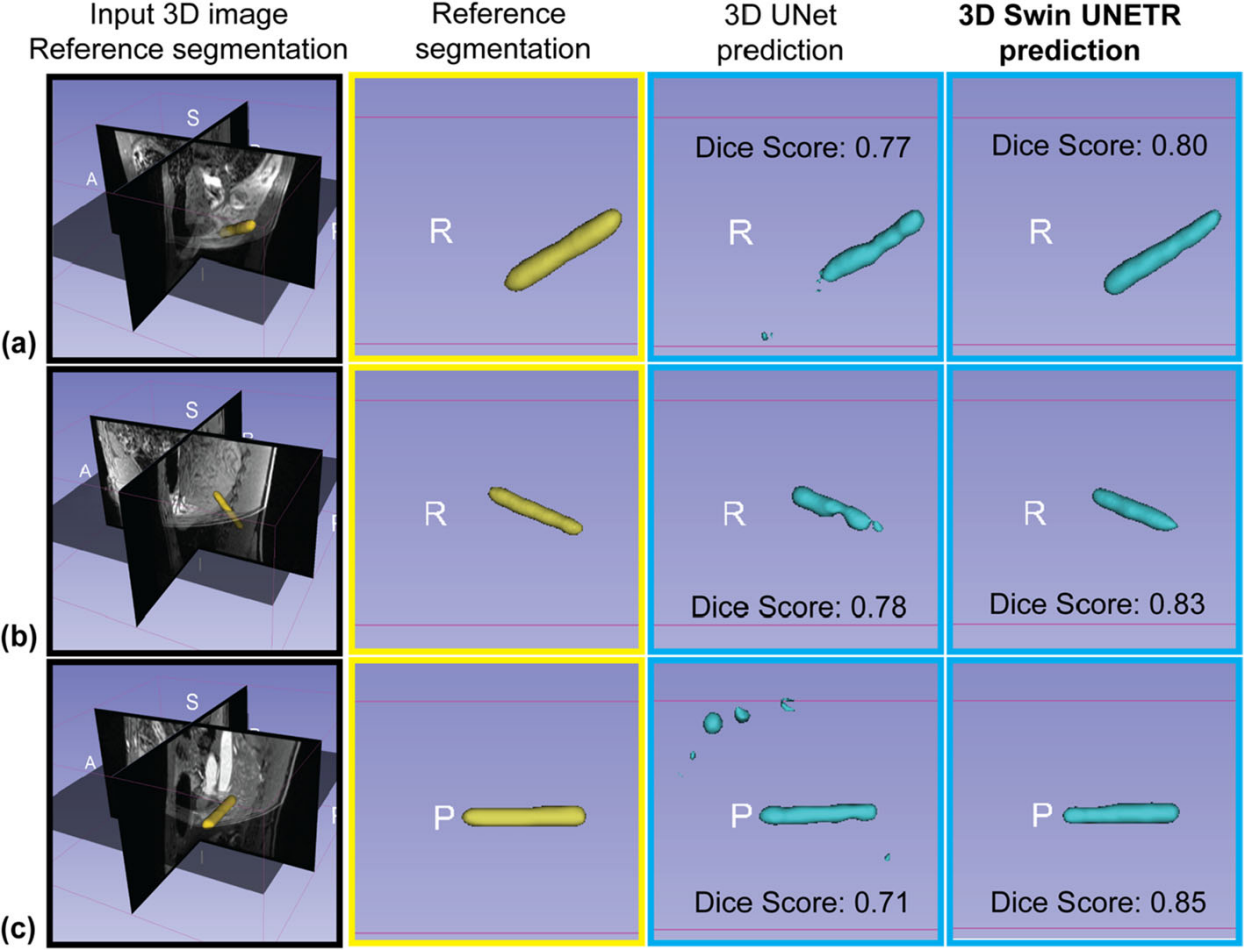
Examples of 3D needle feature segmentation outputs before applying the false-positive removal module. 3D needle feature segmentation references (yellow) and neural network predictions of 3D needle feature segmentation (blue) generated by 3D Swin UNETR and 3D UNet are shown. **a** The two networks achieved similar Dice scores. **b** 3D UNet resulted in under-segmentation, while 3D Swin UNETR achieved better performance. **c** 3D UNet resulted in over-segmentation, while 3D Swin UNETR achieved better performance

To assess the benefits of pre-training and data augmentation, we performed an ablation study of different training strategies, and the results are summarized in Supplementary Table S1. The average inference time on one NVIDIA RTX A6000 GPU card (48 GB GPU memory) was 2.14 s per 3D volume for 3D Swin UNETR and 2.67 s per 3D volume for 3D UNet. Representative 3D needle feature segmentation results from 3D Swin UNETR and 3D UNet are shown in Fig. 5. The performance of 3D UNet and 3D Swin UNETR were similar in some cases, while more over-segmentation and under-segmentation were observed in 3D UNet segmentation results. The median [interquartile range (IQR)] of Dice scores was 0.80 [0.11] for 3D Swin UNETR and 0.76 [0.10] for 3D UNet (*p* = 0.0073).

**Fig. 6:**
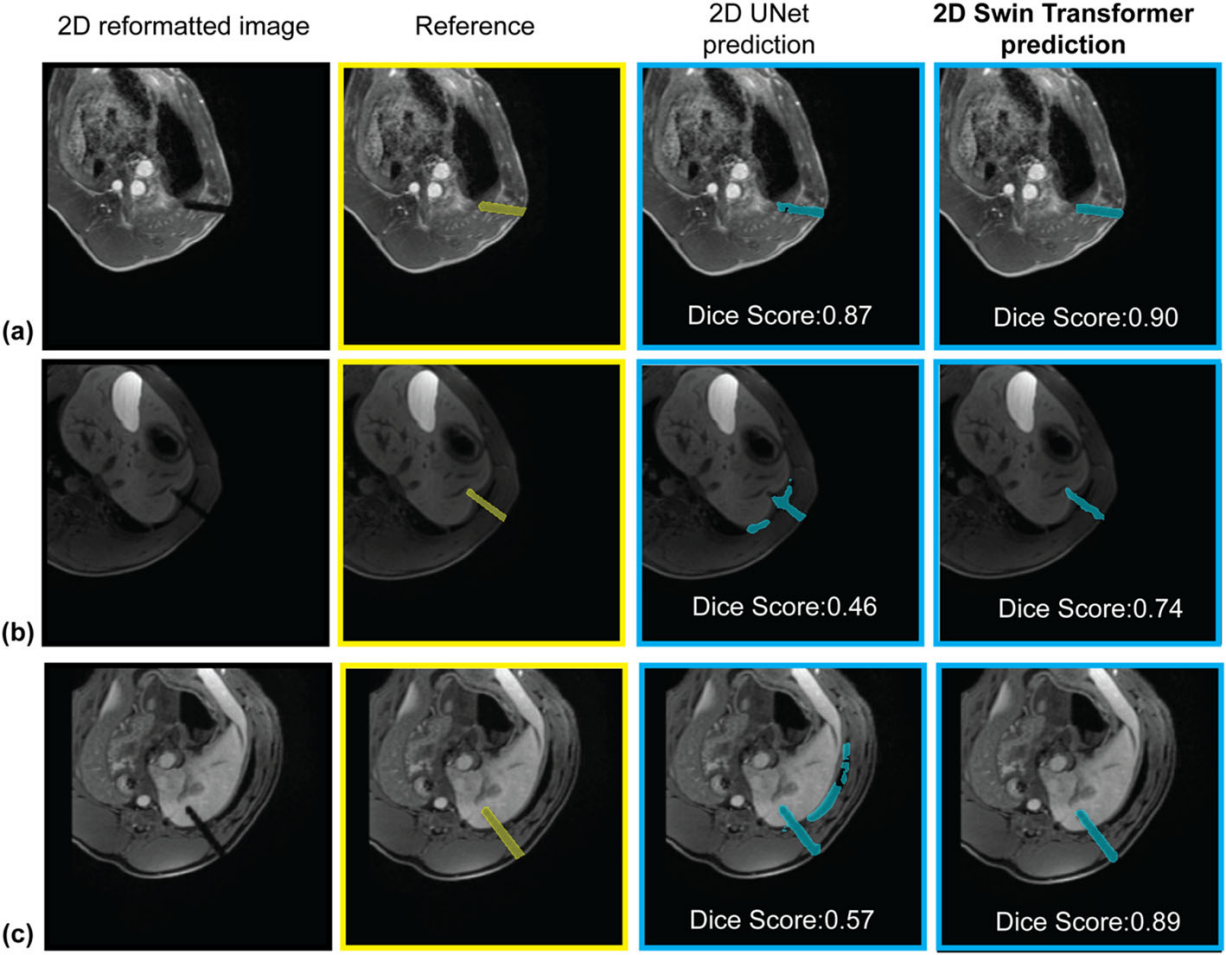
Examples of 2D needle feature segmentation. The input 2D reformatted image, 2D needle feature segmentation references (yellow), and neural network predictions of 2D needle feature segmentation (blue) generated by 2D Swin Transformer and 2D UNet are shown. **a** The two networks achieved similar Dice scores. **b** 2D UNet resulted in under-segmentation and over-segmentation, while 2D Swin Transformer achieved better performance. **c** 2D UNet resulted in over-segmentation, while 2D Swin Transformer achieved better performance

For 2D needle feature segmentation on 2D reformatted images, representative outputs of 2D Swin Transformer and 2D UNet are shown in Fig. 6. The average inference time on the same GPU was 0.011 s per 2D image for the 2D Swin Transformer and 0.016 s per 2D image for the 2D UNet. The median [IQR] of Dice scores was 0.93 [0.04] for 2D Swin Transformer and 0.90 [0.14] for 2D UNet (*p* = 0.0110).

**Fig. 7:**
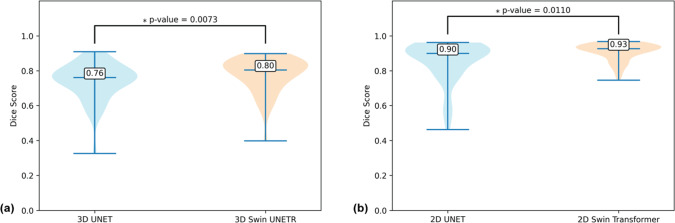
Needle feature segmentation Dice scores from cross-validation (49 sets of 3D MRI). **a** Violin plots of the Dice scores for 3D needle feature segmentation using 3D UNet and 3D Swin UNETR. **b** Violin plots of the Dice scores for 2D needle feature segmentation using 2D UNet and 2D Swin Transformer. The numbers shown on the violin plots are the medians of the Dice scores. In the pair-wise comparisons, *p*-values of the Wilcoxon signed rank test are shown on the connecting lines. * indicates *p* < 0.05

These results (Fig. 7) show statistically significant differences between the performance of 3D Swin UNETR and 3D UNet for 3D needle segmentation, and between the performance of 2D Swin Transformer and 2D UNet for 2D needle segmentation. These results provide evidence that the Swin Transformer-based networks outperform the UNet-based networks in 3D and 2D needle feature segmentation for our application with a limited training dataset.

### 3D needle localization

**Fig. 8:**
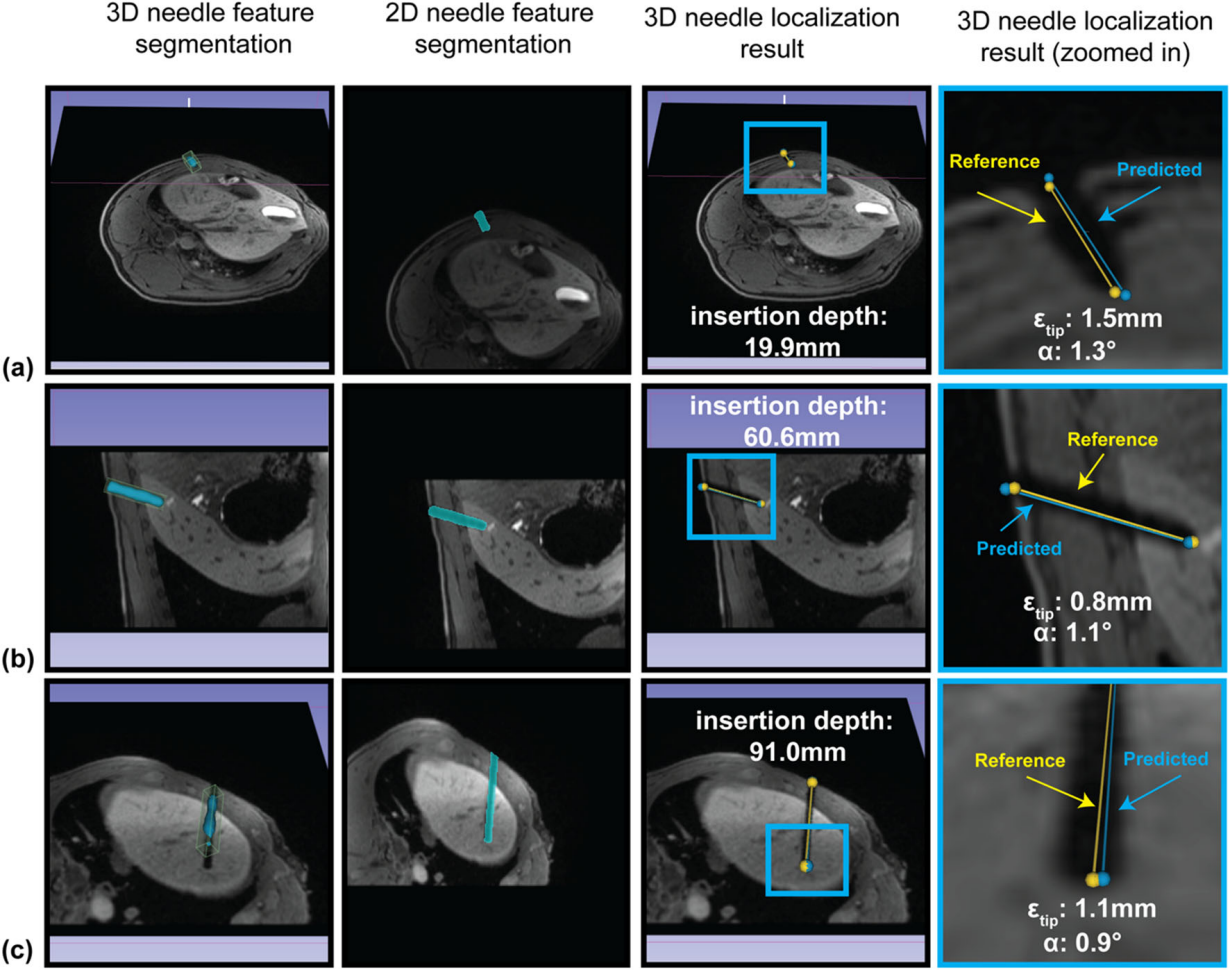
Example outputs from the proposed 3D needle localization pipeline. **a** Shallow insertion depth around 20 mm. **b** Moderate insertion depth around 60 mm. **c** Deeper insertion depth around 90 mm. 3D needle feature segmentation: 3D needle feature segmentation shown with the 2D reformatted image plane in 3D Slicer. 2D needle feature segmentation: 2D needle feature segmentation shown on the 2D reformatted image. 3D needle localization results: Predicted (blue) and reference (yellow) needle tip and axis in 3D space. The needle tip error (${\varepsilon }_{tip}$; mm) and needle axis error ($\alpha$; deg) are reported for each example

The range of needle insertion depth was 1.94–12.26 cm, which is comparable to the skin-to-target length observed in clinical MRI-guided interventions in human subjects (approximately 2–18 cm) [41, 42]. The range of needle insertion angle (angle between the needle and axial plane) was −87.64° to 2.23°. The end-to-end computational time of 3D needle localization was about 6 s per 3D volume for the proposed pipeline and about 4 s for the pipeline without the 2D network. Figure 8 shows example outputs of the pipeline. Volume-rendered displays of the pipeline outputs are shown in Supplementary Video S1.

**Fig. 9:**
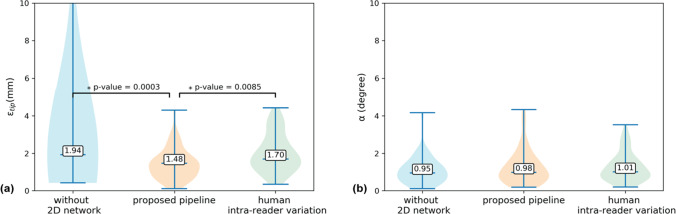
Automatic 3D needle localization results from cross-validation (49 sets of 3D MRI). **a** Violin plots of needle tip localization error (${\varepsilon }_{tip}$) and **b** violin plots of needle axis localization error ($\alpha$) of the proposed pipeline, pipeline without 2D network, and human intra-reader variation. The numbers shown on the violin plots are the medians of the results. In the pair-wise comparisons, p-values of the Wilcoxon signed rank test are shown on the connecting lines. * indicates *p* < 0.05

Figure 9 shows the 3D needle localization results of the proposed pipeline and pipeline without the 2D network (step 1 only) compared with human intra-reader variation as measured by ${\varepsilon }_{tip}$ and $\alpha$. The ${\varepsilon }_{tip}$ of the proposed pipeline had a median of 1.48 mm (1.09 pixels) and was smaller than the pipeline without the 2D network (median of 1.94 mm; *p* = 0.0003, Wilcoxon signed rank test) and human intra-reader variation (median of 1.70 mm; *p* = 0.0085, Wilcoxon signed rank test). There were no significant differences (*p* = 0.5043, Kruskal–Wallis test) in $\alpha$ between the proposed pipeline (median of 0.98°), the pipeline without the 2D network (median of 0.95°), and human intra-reader variation (median of 1.01°).

## Discussion

In this study, we developed a coarse-to-fine automatic deep learning-based pipeline for 3D needle localization on intra-procedural 3D MR images. We used datasets obtained from in vivo MRI-guided interventions in pig livers. The anatomical similarity between pig and human livers is crucial for ensuring that the needle localization pipeline's development and testing are relevant for future translation to clinical applications in human patients. The proposed pipeline achieved accurate 3D needle localization with a median needle tip localization error of 1.48 mm (1.09 pixels) and a median needle axis localization error of 0.98°. This level of accuracy is sufficient for interventions in the liver (e.g., biopsy or ablation) since clinically relevant lesions typically have a diameter of at least 5–10 mm [41, 43]. With an end-to-end computational time of about 6 s, the proposed pipeline shows the potential to accelerate the current step-and-shoot MRI-guided needle intervention workflow, which involves manual 3D needle localization steps that each take several minutes.

For 2D and 3D needle feature segmentation, we adopted 2D Swin Transformer and 3D Swin UNETR, respectively. The statistical analyses showed that 3D Swin UNETR and 2D Swin Transformer outperformed the 3D UNet and 2D UNet, which was consistent with the findings of other studies that compared Swin Transformer and UNet-based networks for biomedical image segmentation tasks [39, 44, 45]. These results demonstrated the advantage of the Swin Transformers in capturing global information when segmenting a small object (i.e., the needle) in a large FOV with complex anatomical structures.

We compared the performance of the proposed pipeline and the pipeline without the 2D network. Under- or over-segmentation of the 3D Swin UNETR still existed due to the limitation of the size of the 3D MRI training dataset. The under- or over-segmentation usually appeared near the needle tip and entry points and therefore had little effect on the needle axis localization but could lead to large needle tip localization errors in the pipeline without a 2D network. Therefore, combining the 2D network in the pipeline was necessary to compensate for the under- or over-segmentation result of the 3D Swin UNETR. In the future, the 2D network might become unnecessary if the 3D network achieves the required accuracy for guiding interventions with additional training data.

There were limitations to this study. Firstly, due to the limited size of the intra-procedural 3D MRI dataset, the training of the networks was affected, and all the results reported here were from cross-validation experiments. In the future, more interventional experiments will be conducted to acquire more data. The additional data will expand the training dataset and enable independent testing for a more comprehensive assessment of the pipeline's performance. Secondly, the reference of the needle tip and axis was annotated by one observer with a washout period of two weeks to assess the human intra-reader variation. Future work can consider multiple observers and use majority voting for needle tip localization reference creation. Thirdly, inline deployment and prospective demonstration of the proposed pipeline in the context of a procedure was not yet achieved. Future work will focus on integrating and testing the proposed pipeline in in vivo MRI-guided interventions.

## Conclusion

In this work, we developed a deep learning-based pipeline for automatic 3D needle localization on intra-procedural 3D MR images. The pipeline had a coarse-to-fine structure where it adopted 3D Swin UNETR for initial segmentation of the 3D needle feature and 2D Swin Transformer for fine segmentation of the needle feature in the 2D reformatted image plane. The proposed pipeline achieved rapid and accurate 3D needle localization within the range of expert human performance and thus has potential to improve MRI-guided percutaneous interventions.

## Supplementary Information

Below is the link to the electronic supplementary material.

Supplementary file1 (DOCX 34 KB)

Supplementary file2 (MP4 44567 KB)
