# Supplementary material for: Deep learning-based automatic pipeline for 3D needle localization on intra-procedural 3D MRI
Source: Int J Comput Assist Radiol Surg. 2024 Mar 23;19(11):2227–37. doi: 10.1007/s11548-024-03077-3 (PMC11541278; doi:10.1007/s11548-024-03077-3)
Supplement: Supplementary file 1 — Supplementary file1 (DOCX 34 KB) [file 11548_2024_3077_MOESM1_ESM.docx]

**SUPPLEMENTAL MATERIALS**

|  | **Pre-training** | **Data augmentation** | **Dice score: Median (IQR)** |
| --- | --- | --- | --- |
| **3D Swin UNETR** | ✓ | ✓ | **0.80 (0.11)** |
|  | ✓ |  | 0.56 (0.40) |
|  |  | ✓ | 0.76 (0.22) |
| **2D Swin Transformer** | ✓ | ✓ | **0.93 (0.04)** |
|  | ✓ |  | 0.91 (0.03) |
|  |  | ✓ | 0.90 (0.05) |

**Supplementary Table S1:** Ablation study of the effects of pre-training and data augmentation. IQR: interquartile range.

[*Video uploaded separately*]

**Supplementary Video S1:** The volume-rendered displays of the pipeline outputs corresponding to **Fig. 8**. The video shows the initial 3D needle feature segmentation result from 3D Swin UNETR, the 3D needle feature segmentation result and the bounding box overlaid on the 2D reformatted image, and the predicted needle tip and axis overlaid on the 2D reformatted image in a rotating 3D view.
